# “*Debaryomyces subglobosus*” – Management of a rare case of fungal septic arthritis post arthroscopic rotator cuff repair – a case report

**DOI:** 10.1051/sicotj/2021057

**Published:** 2021-11-29

**Authors:** Aizel Sherief Palasseril, Sathya Vignes Gopinath, Ramakrishnan Thanikachalam, Sanjoo Anto Prabhu, Vivek Rajamanoharan, Mymoonah Risha Shahul

**Affiliations:** 1 Department of Orthopaedics, Parvathy Ortho Hospital Chennai 600044 India; 2 Department of Plastic Surgery, Parvathy Ortho Hospital Chennai 600044 India; 3 Department of Microbiology, Sree Balaji Medical College & Hospital Chennai 600044 India

**Keywords:** Shoulder, Arthroscopy, Rotator cuff repair, Uncontrolled diabetic, Fungal Septic Arthritis

## Abstract

Fungal septic arthritis of the shoulder is extremely rare in post arthroscopic rotator cuff repair patients. We report our experience in successfully identifying and managing a rare case of fungal septic arthritis with *Debaryomyces subglobosus* in an uncontrolled diabetic patient who underwent arthroscopic rotator cuff repair in 2019. Our patient had complete resolution of symptoms and a reasonable functional recovery within 2 months of debridement and initiation of the specific anti-fungal. This case highlights the importance of high clinical suspicion for atypical fungal infections, and the use of culture-independent modern diagnostic tools like DNA-PCR as adjuncts to successfully identify rare pathogens in immunodeficient patients presenting with vague, nonspecific symptoms of infection.

## Introduction

The incidence of deep-rooted infections after arthroscopic rotator cuff repair is around 0–3.4% [1]. Infection rates are higher when combined mini-open procedures are done, and most of the causative organisms are normal skin flora like *Staphylococcus aureus, Staph epidermidis, Propionibacterium acnes*, and *Corynebacterium* [2]. Atypical infections have been reported with rare organisms like *Pseudomonas aeruginosa*, *Actinomyces* species, *Mycobacterium tuberculosis, Nontubercular mycobacteria*, and fungi, especially in immunodeficient patients [3, 4, 12]. Fungal septic arthritis after shoulder surgery is rare and often poses diagnostic and treatment challenges [5]. Very few cases have been reported so far in the western literature, and the mainstay of fungal infections are from the Candida and Aspergillosis family [5, 13] (Table 1).

**Table 1:** Rare organisms infecting operated joints.

1. Actinomyces’ Species [2]
2. Pseudomonas aeruginosa [3]
3. Non-tubercular mycobacteria [4]
4. Mycobacterium tuberculosis [12]
5. Aspergillosis and Candida [5, 13]
6. Maduralla mycetoma [14]

Here we report a case of septic arthritis of the shoulder with a relatively novel rare fungus – *Debaryomyces subglobosus* [6], in an uncontrolled diabetic, immunodeficient, arthroscopic rotator cuff repair patient. Modern diagnostic tools like DNA-PCR have helped us in detecting such rare organisms. The genus *Debaryomyces* was first reported by Klocker in 1909, and a major description of its species was done years later by Lodder and Kreger-van Rij in 1952 [7]. Very rarely, a different specie *Debaryomyces hansenii* have been reported to infect human intestines in patients with inflammatory bowel disease [8]. By far, we have not been able to find any literature on this genus, being the reason for septic arthritis in humans. They are often found in soil, water, indoor air, and processed food. Earlier, this fungus was thought to be *Candida flareri* until recently when they were confirmed as separate species under *Debaryomyces* by rRNA gene intergenic spacer fingerprinting [9, 10]. Historically the diagnosis and treatment of fungal septic arthritis have been challenging, especially in immunodeficient patients, and often resulted in poor functional outcomes, owing to the long course of infection and delay in diagnosis and treatment.

## Case report

**Figure 1:**
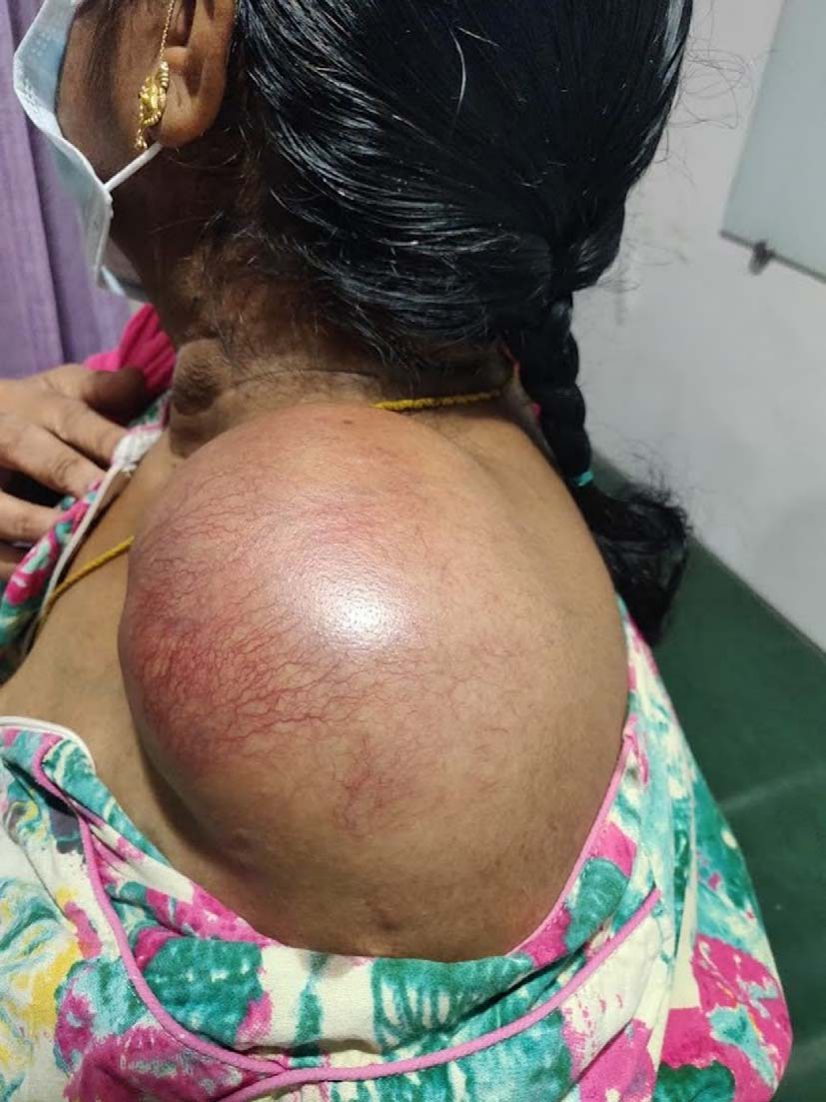
Clinical photograph of swelling over left shoulder.

A 52-year-old female from a remote village in southern India, who is a known case of uncontrolled type 2 diabetes and hypothyroidism for the past 5 years, presented with a 1-year history of pain over her left shoulder and 2 months of increasing swelling of the same shoulder after a trivial fall. She had undergone an arthroscopic rotator cuff repair for the same side in 2019, with metal and bioabsorbable suture anchors from elsewhere. After the surgery, she was doing well with her shoulder, except for the occasional pain, that aggravated to the present status in 2 years. Owing to the COVID-19 pandemic, she did not have regular post-operative follow-ups. In March 2021, she had a trivial fall over the same shoulder, and since then, she noticed an increasing swelling around the same shoulder, which grew from the size of a lemon to a half-cut watermelon spanning in between her neck and affected shoulder (Figure 1).

**Figure 2:**
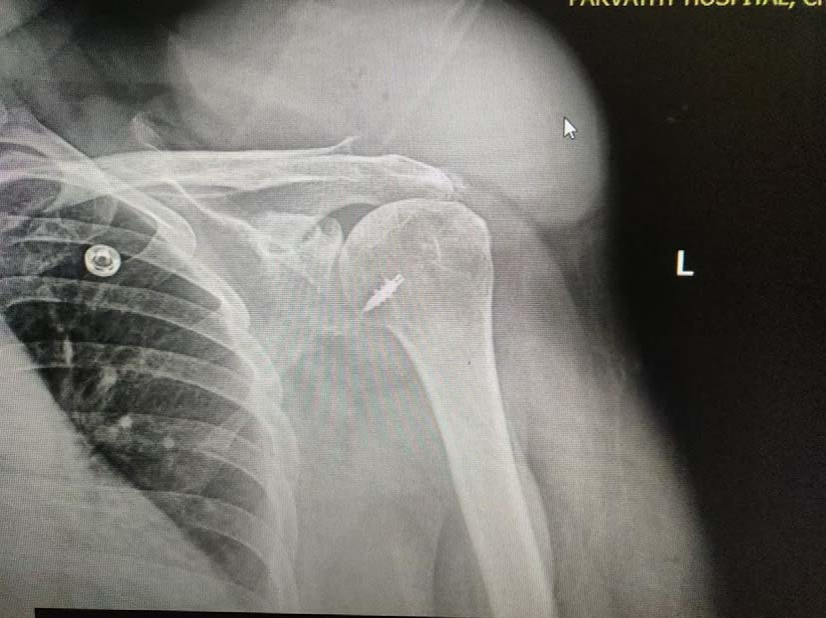
X-ray of the affected shoulder showing destruction of AC joint and metal anchor in-situ.

**Figure 3:**
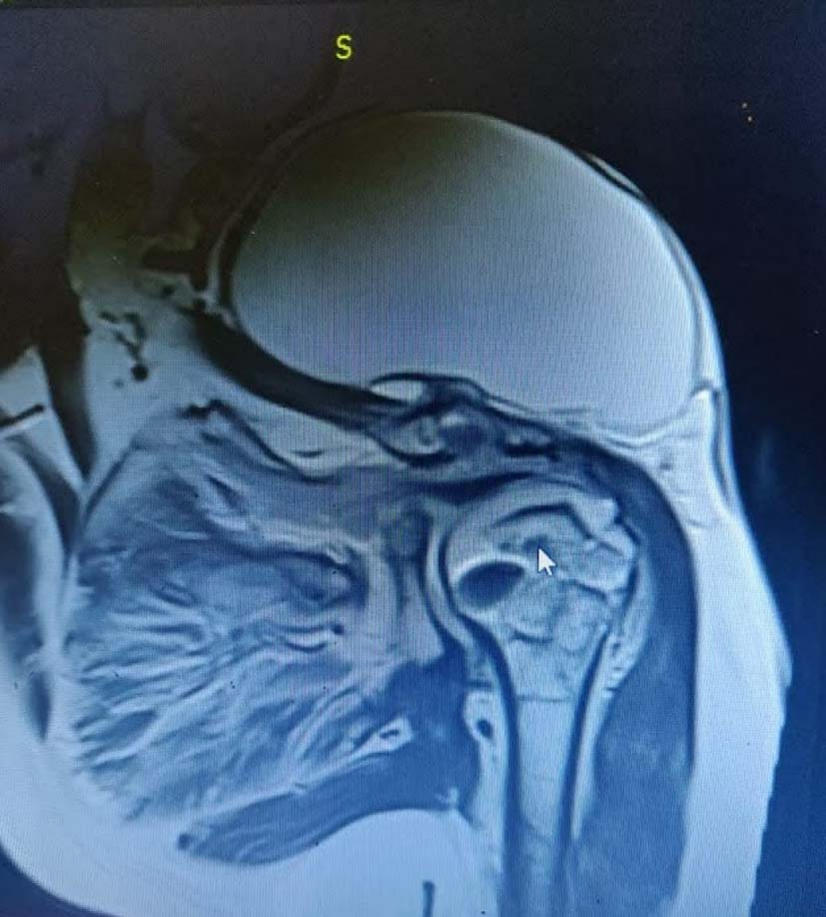
T2 weighted coronal MRI image of the shoulder showing the subcutaneous sac communicating to AC and Shoulder Joint.

Owing to the raging COVID situations in India, she could not attend a specialist hospital and took treatment at a local bonesetter with herbal massages until she presented here. She also had a few courses of broad-spectrum antibiotics from local hospitals with no resolution of her symptoms. The swelling looked fiery red at the time of presentation and had a local rise in temperature, and her shoulder movements were painful. There was no distal neurovascular deficit. There was no history of fever, chills or rigour, loss of weight or loss of appetite, or evening rise of temperature, or involvement of any other joints. There was no past or contact history of Tuberculosis (TB) nor any history of recent COVID infection. The pre-operative evaluation revealed normal leukocyte and neutrophil counts but raised C-reactive protein (CRP) – 67.6 and a normal erythrocyte sedimentation rate (ESR) – 06 mm. Her Hba1c was 10 depicting her uncontrolled diabetic status. Her blood culture was negative. An X-ray revealed bony destruction at the lateral end of the clavicle with the metal anchor in situ in the shoulder (Figure 2). Magnetic resonance imaging (MRI) revealed a large fluid collection with few septations in the subcutaneous tissue in the supraclavicular region communicating to the acromioclavicular (AC) joint, which was widened and eroded. Moderate glenohumeral joint effusion extending into the subacromial and subdeltoid spaces were also reported suggestive of corresponding bursitis (Figure 3). Our differential diagnosis where pyogenic/tubercular/fungal septic arthritis, which are the usual afflictions.

**Figure 4:**
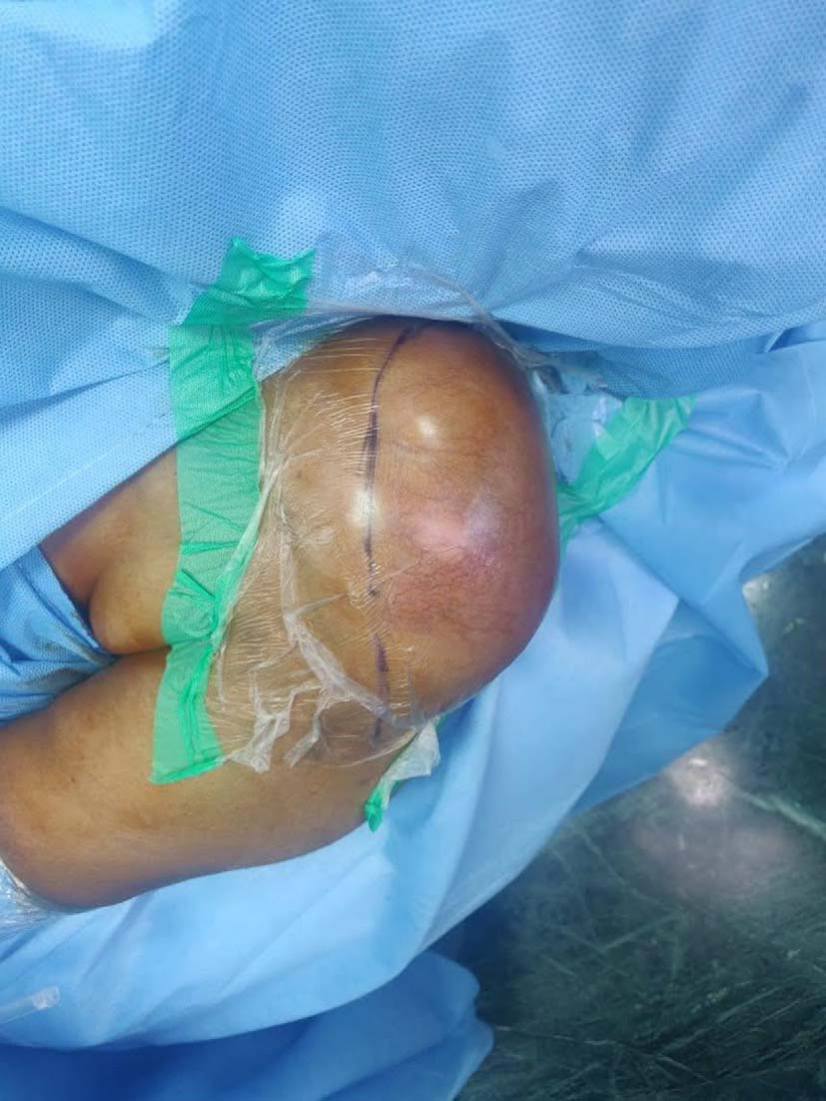
Surgical incision.

**Figure 5:**
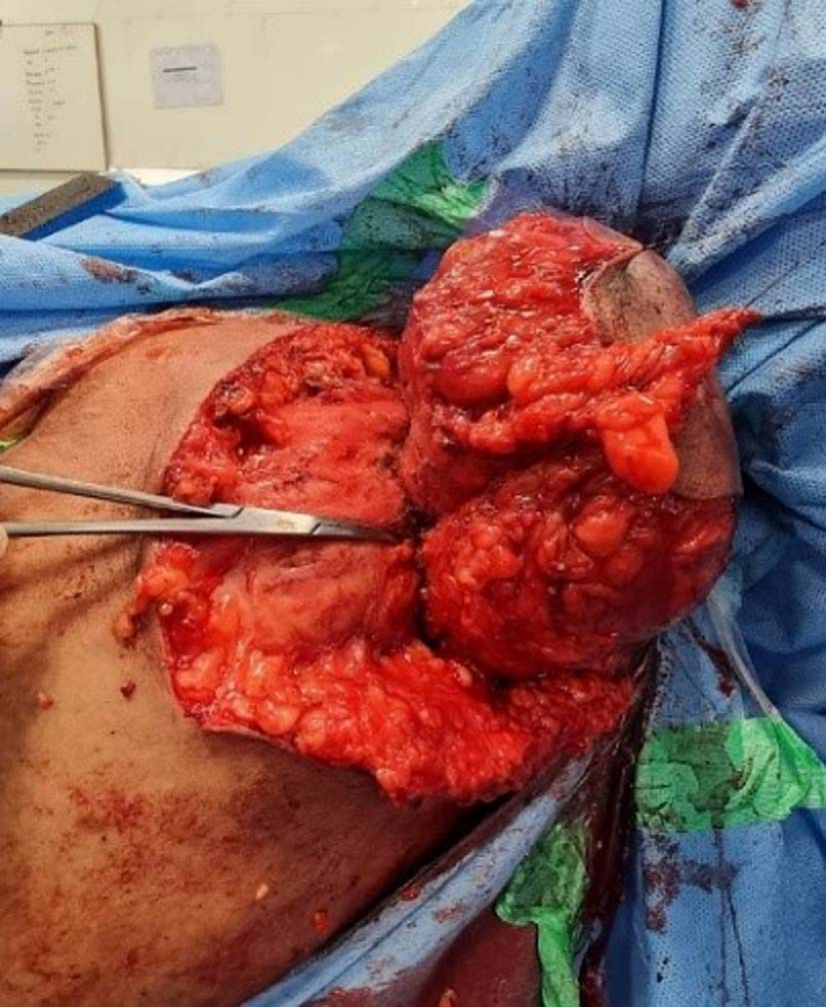
Sac with its communication to AC joint shown.

**Figure 6:**
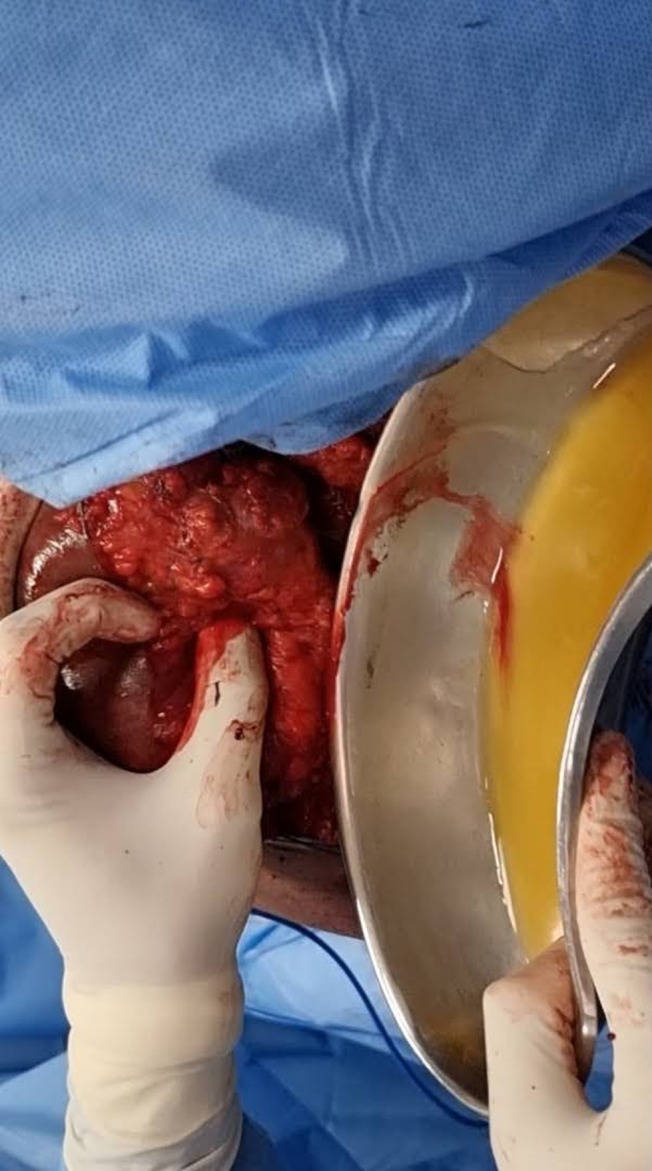
Straw yellow coloured pus drained out.

**Figure 7:**
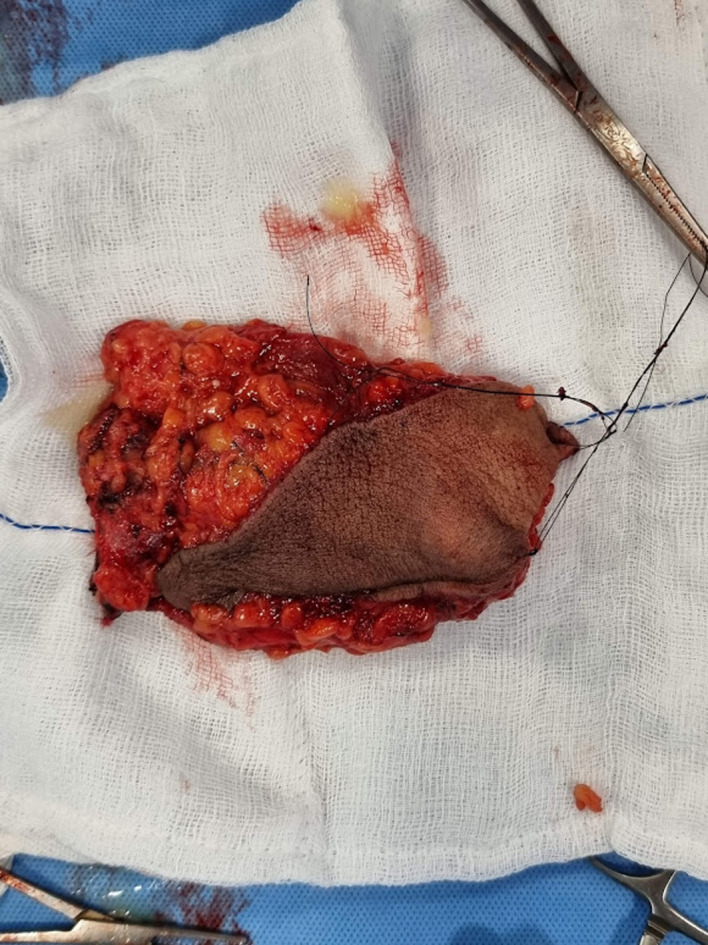
Sac excised in toto with superficial skin.

**Figure 8:**
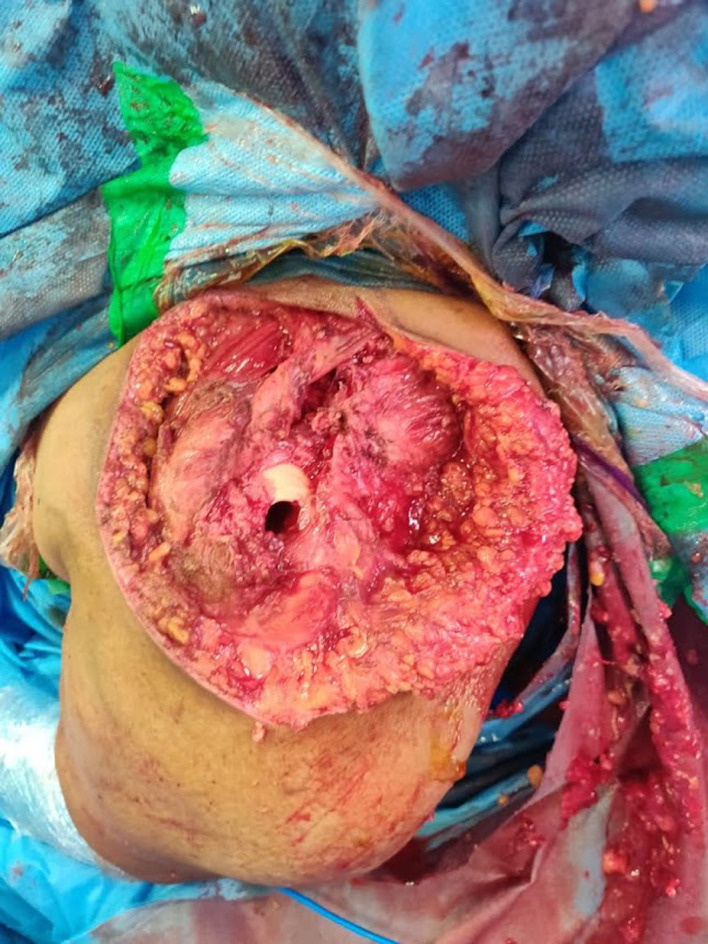
*Osteomyelitic nidus* retrieved from the humeral head through the debridement hole.

The patient underwent swelling excision in toto and debridement of the left acromioclavicular and shoulder joint (Figures 4, 5, and 7). Surprisingly the whole sac of swelling was traced from the subcutaneous plane through the AC joint to the subacromial and subdeltoid bursae up to the humeral head entry site for the metal anchor. Around 500 mL of straw yellow pus was drained out of the excised sac and from the shoulder joint (Figure 6). The deep-seated metal anchor in the humeral head was surrounded by a nidus of osteomyelitis sequestrum, which was debrided and retrieved along with the metal and bio absorbable anchors (Figure 8).

**Figure 9:**
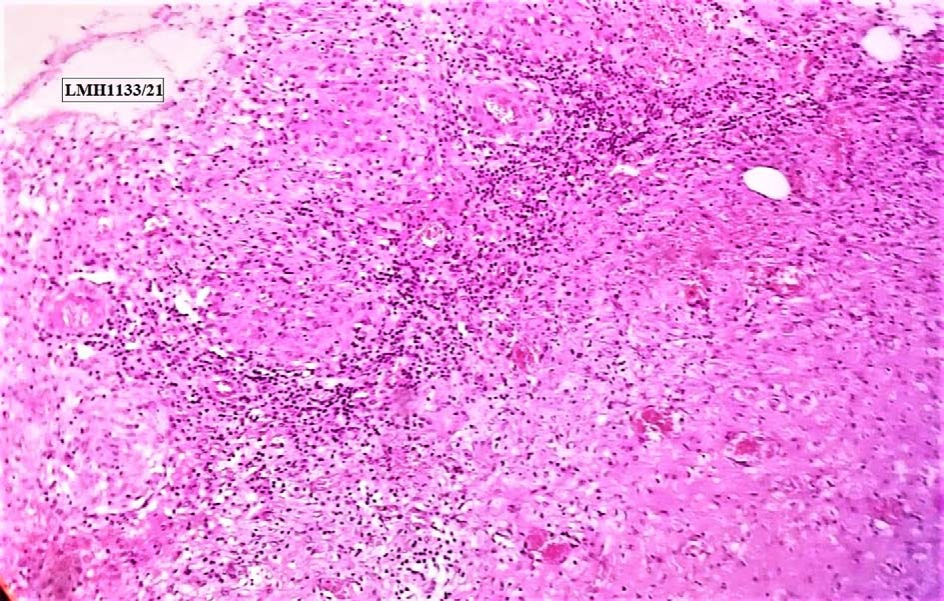
Hand E-stained slide [×100 magnification] showing multinucleated giant cells and granulation tissue.

**Figure 10:**
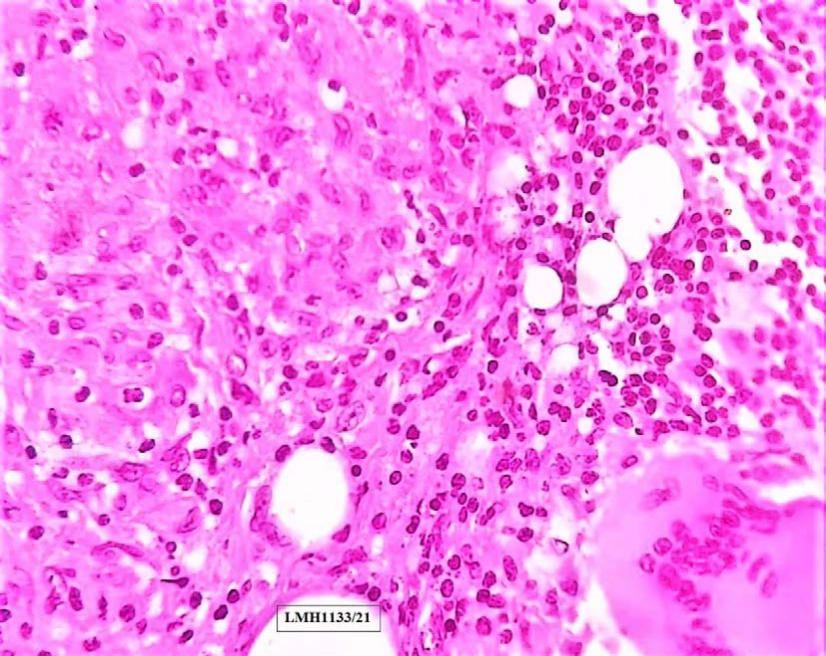
Hand E-stained slide [×400 magnification] showing epithelioid cell forming granuloma.

Multiple specimens of infected tissue and pus were sent for histopathology, aerobic and anaerobic bacterial cultures, acid-fast bacteria (AFB), gram staining, TB GeneXpert, and Fungal DNA-PCR. The bacterial and tubercular reports were all negative and eventually, the fungal DNA detection by PCR was positive for *Debaryomyces subglobosus*, an extremely rare fungi to infect the shoulder joint, even in immunocompromised patients. HPE was consistent with chronic granulomatous infection (Figures 9 and 10). The patient was started on Voriconazole [8], the specific anti-fungal for the same and got a complete resolution of her symptoms within 6 weeks of initiation of treatment. The serum markers for infection, CRP and ESR, returned to normal within this period, and her surgical wound healed completely.

However, her functional recovery was lagging behind, even at two months follow-up, owing to the chronicity of the infection and the residual damages it has already made to the rotator cuff muscles and the joint.

## Discussion

Fortunately, infection after arthroscopic rotator cuff repair is very rare. Most patients who develop infections have chronic, systemic, and immunocompromising conditions, such as diabetes mellitus, blood dyscrasia, renal failure, malignancy, malnutrition, and rheumatoid arthritis with a long history of corticosteroid use [11]. Perioperative optimization of patient comorbidities like diabetes, pre-operative antibiotic prophylaxis targeted at normal skin flora and sterile surgical practices have helped prevent common infections. With a growing number of immunocompromised patient’s newer organisms are being reported infecting operated shoulder joints. The spectrum of atypical organisms ranges from actinomyces species and Pseudomonas aeruginosa, to non-tubercular mycobacterium and fungi. Whenever deep-seated infections are suspected, thorough surgical debridement and long course organism-specific antibiotics are warranted.

The frequency of invasive fungal infections in immunocompromised patients is rising, and the spectrum of pathogens has gone beyond *Aspergillus* and *Candida*. Early, rapid and accurate identification of the pathogenic fungi is of prime importance, for timely initiation of the specific anti-fungal, for better patient outcomes and epidemiological purposes. Culture-independent methods like PCR has high specificity and sensitivity in detecting viable and non-viable fungi. This case highlights the importance of high clinical suspicion for atypical fungal infections like *Debaryomyces* and their highly indolent course of clinical progression in immunodeficient arthroscopic rotator cuff repair patients. The mystery behind infection with such a rare fungus remains unresolved and highlights the importance of adequate perioperative diabetic control, having proper standards in prepping and draping of patients, and use of standard sterilization techniques for instruments used during arthroscopic surgeries [12]. Interestingly after the index rotator cuff repair procedure, our patient had an uneventful immediate post-op, with good surgical wound healing and an excellent functional recovery as well, which deteriorated through a long span of 2 years. However, accurate diagnosis of this fungus using DNA-PCR has helped in timely detection and initiation of the specific anti-fungal, which eventually led to complete resolution of the disease.

## Conclusion

With the growing number of immunodeficient post-operative patients worldwide, it’s high time that we rejig our list of causative pathogens to include atypical microorganisms and use modern culture-independent diagnostic tools like DNA-PCR as adjuncts in diagnosis.

## Conflict of interest

The authors report no conflict of interest.

## Funding

No Funding was obtained from any Individual/ Institution/ Organization.

## Ethical approval

Ethical approval was obtained.

## Informed consent

Written informed consent was taken from the patient.

## Authors contributions

Aizel Sherief Palasseril, Sathya Vignes Gopinath, Ramakrishnan Thanikachalam – Conceptualization, data curation, investigation, resources, software, writing original draft, writing-reviewing and editing.

Vivek Rajamanoharan, Sanjoo Anto Prabhu, Mymoonah Risha Shahul – Conceptualization, resources, data curation.
